# The European Bioinformatics Institute’s data resources 2014

**DOI:** 10.1093/nar/gkt1206

**Published:** 2013-11-23

**Authors:** Catherine Brooksbank, Mary Todd Bergman, Rolf Apweiler, Ewan Birney, Janet Thornton

**Affiliations:** European Molecular Biology Laboratory, European Bioinformatics Institute, Wellcome Trust Genome Campus, Hinxton, Cambridge CB10 1SD, UK

## Abstract

Molecular Biology has been at the heart of the ‘big data’ revolution from its very beginning, and the need for access to biological data is a common thread running from the 1965 publication of Dayhoff’s ‘Atlas of Protein Sequence and Structure’ through the Human Genome Project in the late 1990s and early 2000s to today’s population-scale sequencing initiatives. The European Bioinformatics Institute (EMBL-EBI; http://www.ebi.ac.uk) is one of three organizations worldwide that provides free access to comprehensive, integrated molecular data sets. Here, we summarize the principles underpinning the development of these public resources and provide an overview of EMBL-EBI’s database collection to complement the reviews of individual databases provided elsewhere in this issue.

## INTRODUCTION

The molecular life sciences are becoming increasingly data-driven and reliant on open-access databases (1). This is as true of the applied sciences as it is of fundamental research: in the past year, we have witnessed announcements that the UK’s National Health Service will invest in sequencing the genomes of up to 100 000 citizens (see http://www.gov.uk/government/speeches/strategy-for-uk-life-sciences-one-year-on and http://news.sciencemag.org/biology/2012/12/u.k.-unveils-plan-sequence-whole-genomes-100000-patients); the Faroe Islands are planning to sequence the genome of every citizen who wishes to have this information (see http://www.fargen.fo/en/), and large-scale metagenomics projects are helping us to map the global biodiversity of the oceans (2).

The European Bioinformatics Institute (EMBL-EBI), part of the European Molecular Biology Laboratory, makes these large-scale efforts possible. It helps scientists deposit their research data into public collections, produces value-added knowledge bases and makes its entire holdings accessible to all, thereby enabling millions of scientists worldwide to explore, analyse, interpret and derive new knowledge from decades of scientific endeavour.

Among its other roles (Appendix 1), EMBL-EBI has a mission to provide free and open access to biomolecular information, spanning scientific literature and the data supporting it: DNA and protein sequences; biomolecules and their structures, functions, reactions and interactions; and practical tools for analysis and discovery.

These offerings include personally identifiable genetic and phenotypic data resulting from biomedical research projects—an area of growing importance as healthcare systems embrace genomic medicine. Managing access to these data sets is a high-priority activity at EMBL-EBI.

EMBL-EBI’s core resources are foundational members of international consortia, which share data globally and foster competitiveness among their members. Some of these collaborations have a long history [e.g. the International Nucleotide Sequence Database Collaboration (INSDC) (3), the worldwide Protein Data Bank (wwPDB) (4), UniProt (5) and Ensembl (6)]. Others, driven by EMBL-EBI, are more recent [e.g. IMEx (7) for protein interaction data; ProteomExchange (8) for protein identification data and COSMOS (9) for metabolomics data]. The Global Alliance (10)—a large-scale international effort to enable the secure sharing of genomic and clinical data—is the most recent of these. Each of these collaborations exemplifies the fundamental principles of EMBL-EBI service provision (Appendix 2).

## DESIGNED TO BE USED

EMBL-EBI embraces user-centred design (UCD), an approach that focuses on the behaviour and needs of the people who will actually use the product. UCD has been successfully applied to design in many different domains, although its application to bioinformatics services (11) is relatively recent. The case for using UCD for bioinformatics services is compelling: even major bioinformatics resources are known to suffer from usability problems (12), which prevent users from completing tasks (13).

By placing the user at the forefront of our minds as we design, test and implement our services, we create more useful and user-friendly resources. This approach has been used to completely redesign the EMBL-EBI website—a major project that has involved every team at EMBL-EBI. The redesign puts users at the centre of the process, providing an intuitive new interface to EMBL-EBI services. It aims for consistent functionality without stifling the individual data resource brands.

EMBL-EBI’s search engine (14) displays results in an organized manner, according to the central dogma of molecular biology (i.e. DNA makes RNA makes protein). This results in an uncluttered results ‘dashboard’ from which users can explore genes, protein sequences, gene expression, molecular structures and related scientific literature. The search allows easy comparison of key information for human, mouse, fly and other species.

**Figure 1.**
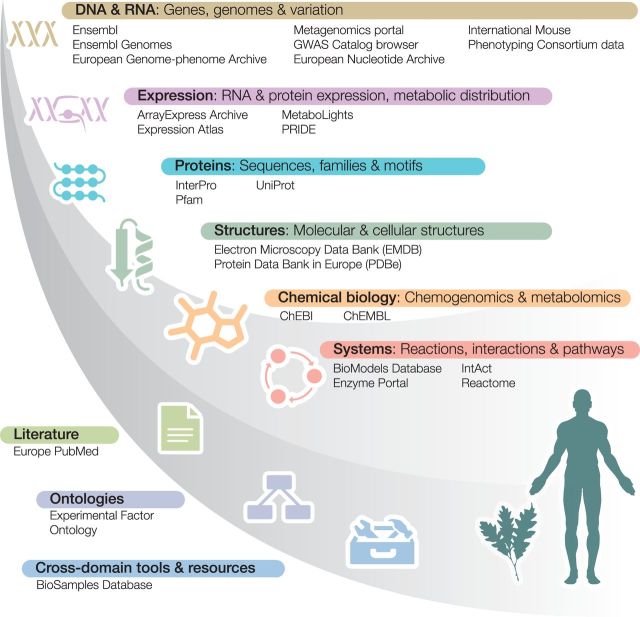
EMBL-EBI’s core data resources. The figure summarizes the resources described in this review; a fuller summary of EMBL-EBI’s data resources and tools, which links to each resource, is available at http://www.ebi.ac.uk/services.

Bioinformatics services on the EMBL-EBI website are displayed according to nine major themes (Figure 1; see also http://www.ebi.ac.uk/services), which were informed by user feedback. We have organized this review in the same way.

## LITERATURE

Access to the scientific literature is a basic requirement for research. EMBL-EBI coordinates the development of Europe PubMed Central Europe (PMC) (15) in collaboration with The University of Manchester (Mimas and NaCTeM) and the British Library. PMC is part of PMC International, which is coordinated by the US National Center for Biotechnology Information and includes PMC Canada. Launched in November 2012, Europe PMC is funded through a collective of European funders, coordinated by the Wellcome Trust.

Europe PMC combines the entire collection of PubMed abstracts, PMC full-text articles, patent abstracts (European, US and international), National Health Service (NHS) clinical guidelines, Agricola records and other record types, and builds innovative tools to help researchers explore every aspect of the literature. Because it is developed at EMBL-EBI, it is uniquely positioned to link abstracts and articles seamlessly to the underlying data. For example, Europe PMC is integrated with UniProt, the Protein Data Bank in Europe (PDBe) and the European Nucleotide Archive (ENA).

## CROSS-DOMAIN TOOLS AND RESOURCES

The integration of different -omics data types requires the consistent application of metadata to data sets derived from the same sample. Sample metadata are managed within EMBL-EBI’s BioSamples database (16), which provides links to assays for specific samples (including reference samples such as cell lines) and accepts direct submissions. In 2013, the BioSamples database launched a new user interface, application programming interface (API) and submission-accessioning service.

## ONTOLOGIES

Biologists and bioinformaticians look to ontologies and other types of controlled vocabularies as a means of standardizing the way data are described, queried and analysed. Subtle differences in the use of terms and phrases can hamper communication among scientists, and can make automated data exchange prohibitively difficult. Ontologies address these issues, making information in databases more readily human- and computer-readable.

The Gene Ontology (GO) (17) is a major bioinformatics initiative to unify the representation of gene and gene-product attributes across all species. Groups participating in the GO Consortium include major model organism databases and other bioinformatics resource centres. At EMBL-EBI, the GO editors play a key role in managing the distributed task of developing and improving GO, whereas the UniProt GO annotation (GOA) program adds high-quality GOAs to proteins in the UniProt Knowledgebase (UniProtKB) (18). Recent enhancements include expanding the functionality of GO’s direct ontology submission tool, TermGenie, which now includes a ‘free form’ input for experienced users; integration of the GO and ChEBI ontologies (19); and improvements to the electronic GOA pipeline.

Another cross-cutting tool is the Experimental Factor Ontology (EFO) (20), which began its life as a practical means of categorizing gene expression data sets. In the past year, it has broadened its application considerably to support annotation of genome-wide association studies (GWAS) and the integration of genomic and disease data.

## DNA AND RNA

Nucleotide sequence data are a central reference point onto which many other types of information can be built.

### The public record of nucleotide sequence data

The ENA (21) manages the staggering volumes of data generated by next-generation sequencing. The ENA team has developed CRAM, an openly accessible software toolkit and file format for compressing sequence data, leveraging the specific data properties of DNA sequence (22). Officially launched in November 2012, CRAM is a community-led endeavour that is being incorporated into existing tools and pipelines so that researchers can save on local storage space. It also has the advantage of keeping the public archives to a manageable size.

### Reference genomes

Ensembl (6), produced jointly by EMBL-EBI and the Wellcome Trust Sanger Institute, enables and advances genome science by providing high-quality integrated annotation on vertebrate genomes within a consistent and accessible infrastructure. Ensembl’s new features include the Variant Effect Predictor (23), which predicts the effects of variant positions and alleles on overlapping transcripts and regulatory regions. Ensembl features the genomes of 75 vertebrate species [the mountain gorilla (24) being a notable recent addition]. It also houses the substantial data sets produced by the ENCODE project (25).

Ensembl Genomes (26), launched in 2009 to expand EMBL-EBI’s taxonomic coverage of reference genomes, added a significant number of new species to its database in the past year. It now includes the genomes of biting midges, butterflies (27), barley (28), wheat (29) and >6000 bacterial species.

Ensembl Genomes also provides the underlying architecture for several new community portals. Understanding the basis of crop diseases was the driver behind the launch of PhytoPath (http://www.phytopathdb.org), a new portal for plant pathogen data. EMBL-EBI’s involvement in the transPLANT project has spawned a new integrative portal for plant genomics data (http://www.transplantdb.eu). Ensembl Genomes has also made metabolic data for >4000 bacterial genomes available through the Microme portal (http://www.microme.eu).

### Linking genotype to phenotype

EMBL-EBI’s philosophy is to make its data openly available to the research community, but where personally identifiable data are involved, it is important that we honour the consent agreements under which patients provide data, which nearly always exclude the use of genetic data to identify individuals. The European Genome-phenome Archive (EGA) is EMBL-EBI’s service for permanent archiving and sharing of all types of personally identifiable genetic and phenotypic data resulting from biomedical research projects. The EGA contains exclusive data collected from individuals whose consent authorizes data release only for specific research use or to *bona fide* researchers. The EGA provides the necessary security required to control access, maintain patient confidentiality and provide access for those researchers and clinicians who are authorized to view the data. In all cases, data access decisions are made by the appropriate data access-granting organization (DAO) and not by the EGA. An independent Ethics Committee audits the EGA protocols and infrastructure.

Resequencing projects are providing vast amounts of data that link genotype to phenotype, with the ultimate goal of establishing the connections between genetic variation and disease. Two resources reviewed in this NAR database issue provide access to these data, one focusing on GWAS, the other on knockout mice.

EMBL-EBI and the US National Human Genome Research Institute (NHGRI) jointly develop the Catalog of Published GWAS (30). The catalogue is a publicly available manually curated collection of published GWAS with a distinctive and dynamic visualization tool that enables users to click on single-nucleotide polymorphism (SNP)–trait associations mapped to chromosomal locations. Each association is annotated with terms from the EFO (see ‘Cross-domain tools and resources’ above) to help the user identify SNPs associated with a specific phenotype.

The International Mouse Phenotyping Consortium (IMPC) is building the first comprehensive functional catalogue of a mammalian genome (31). To do this, it is creating a knockout mouse strain for every known protein-coding gene—20 000 mouse strains in total—using a rigorously standardized set of phenotyping protocols. These strains will be made available in public repositories, and data pertaining to each will be made publicly available in near real time, along with open tools for their analysis. Project data will be delivered through a service (http://www.mousephenotype.org) managed by the MPI2 consortium (EMBL-EBI, the Wellcome Trust Sanger Institute, MRC Harwell). Users will be able to search by term, gene, tissue or disease, so they may identify associations between phenotype, gene and protocol swiftly. The results are displayed using the same principles and underlying architecture as EMBL-EBI’s global search. The service is expected to launch in early 2014.

### Metagenomics

While the projects described above are accumulating an ever greater depth of knowledge about the genomes of long-studied organisms, another approach—metagenomics (32)—increases ‘breadth’ of knowledge by presupposing nothing about the identity of the organisms present in a sample. EMBL-EBI’s ENA and InterPro teams have created an integrated resource—the Metagenomics Portal (http://www.ebi.ac.uk/metagenomics/)—that allows researchers to submit, archive and analyse genomic information from environments containing many species. New functionality is being added regularly in response to user demand.

## EXPRESSION

The combination of transcriptomics, proteomics and metabolomics data can provide a powerful basis for deriving a system-based understanding of biological systems. To facilitate such integration, EMBL-EBI is working towards the integration of the ArrayExpress Archive (33), Expression Atlas (33), Proteomics Identifications Database (PRIDE) (34) and MetaboLights (35), EMBL-EBI’s newly launched metabolomics database.

EMBL-EBI is developing a Baseline Expression Atlas, which uses high-throughput sequencing-based expression data to report ‘absolute’ gene expression levels, rather than relative levels. Concurrently, significant improvements have been made to the ArrayExpress archive interface, and the resource has accepted its millionth assay.

PRIDE (34) is a public resource for mass spectrometry-based protein expression data. In 2013, PRIDE achieved the successful and stable implementation of the ProteomeXchange data workflow (http://www.proteomexchange.org). As a result, data depositions more than tripled in number of submissions and in volume.

The final database making up this ‘expression and distribution trinity’ is the newly launched MetaboLights (35)—the first general purpose open-access database for metabolomics and its derived information. MetaboLights includes a reference layer with information about individual metabolites, their chemistry, spectroscopy and biological roles, connected with a study archive into which researchers deposit primary and metadata on metabolomics studies.

## PROTEINS

Protein sequence provides another ‘information hub’ for the molecular biologist, onto which experimentally validated information about the behaviour and localization of proteins can be hung, and from which hypotheses about structure and function may be generated.

UniProt (5), the unified resource of protein sequence and functional information, is maintained by EMBL-EBI in collaboration with the Swiss Institute of Bioinformatics and Universities of Georgetown and Delaware. UniProt is closely integrated with Ensembl (6) and Ensembl Genomes (26), and has generated new reference proteome sets to match their genes in the reference genomes. UniProt prioritizes the manual annotation of experimental data for human and other reference proteomes in collaboration with other worldwide resources, ensuring the highest quality knowledge is available to researchers. UniProt’s automatic annotation exploits the results of manual annotation, resulting in a widening of taxonomic and annotation depth.

EMBL-EBI’s protein resources have embraced UCD (see ‘Designed to be used’). The UniProt development team has designed new interfaces that enhance user interaction with the website and facilitate access to data. The UniProt content team has extended the databases to accommodate a rapidly growing volume of data and to incorporate variation and proteomics data.

InterPro (36), EMBL-EBI’s database of protein families, domains and motifs, has completely re-implemented its back-end to optimize user query functionality. A new user interface has been developed and tested with users, and the results have informed the global EMBL-EBI website redesign process.

Pfam (37), a database of hidden Markov models and alignments describing conserved protein families and domains, is one of InterPro’s 11 member databases, and is in the process of migrating from the Wellcome Trust Sanger Institute to EMBL-EBI. Pfam’s latest release adds real-time searches of DNA sequences for matches to Pfam models, representative proteome sequence sets to provide non-redundant views of alignments and annotations to disease.

## STRUCTURES

Understanding molecular structure is crucial to understanding function. PDBe (38,39), the European arm of the worldwide Protein Data Bank collaboration (wwPDB), provides sophisticated tools for analysing structures, several of which were improved significantly in the past year. These include tools that enhance the analysis of nuclear magnetic resonance entries and many improvements to EMDB (38), the European resource for electron microscopy-based models. EMDB now has a new search service and an interactive viewer for electron tomograms. PDBe is increasingly integrated with other types of information, including sequence data [through the SIFTS service (40)] and the GO (17) (through a new module in the PDBeXplore tool).

## SYSTEMS

The genes and gene products encoded by genomes do not act in isolation but do so in coordinated systems, often containing protein, small molecule and oligonucleotide or oligosaccharide components. EMBL-EBI’s molecular systems resources enable researchers to build a holistic view of life at the molecular level, building up from enzymes and their mechanisms, through protein—protein interactions and networks, to pathways and quantitative models.

The Enzyme Portal (41,42), launched in February 2012, combines high-quality data from 10 previously isolated databases and organizes information about each enzyme in such a way that the user can flip from information about a single enzyme function to resolved structures, reactions and pathways, substrates and products, relevance to disease and relevant publications. Users can also search the Enzyme Portal by protein sequence.

IntAct (43), EMBL-EBI’s database of molecular interactions, is now closely integrated with the MINT database at the University of Rome, and is serving as the curation platform for eight global partner organizations based in Canada, India, Ireland, Italy, Singapore, UK and the USA through the IMEx Consortium (7).

EMBL-EBI, the Ontario Institute of Cancer Research and New York University Medical Center jointly develop the Reactome (44,45) database of curated human pathways. Reactome’s website (http://www.reactome.org) has been completely redeveloped, and now features a modular pathway browser, a comprehensive set of web services and integrated molecular-interaction, structural and expression data.

Submissions to the BioModels database (46), EMBL-EBI’s database of computational models of biological processes, have more than doubled since 2011. A new ‘top-down’ approach to building quantitative models has been implemented. Rather than building up from the mechanistic details of a specific process, the new approach uses pathways from data resources such as Kyoto Encyclopedia of Genes and Genomes and Reactome (44) as starting points. The BioModels database is also one of several EMBL-EBI databases that are actively involved in exposing their data to the semantic web: there is now a resource description framework (RDF) representation of the models in the database, and users have access to powerful queries using SPARQL.

## CHEMICAL BIOLOGY

Chemical biology has been a major growth area for EMBL-EBI in the past decade. Major drivers for this growth have included the emergence and maturation of computational systems biology (47) and public investment in computational approaches to drug discovery (48,49).

ChEMBL (50), EMBL-EBI’s database of drugs and bioactive entities, now has a unified chemistry resource lookup and registration system called UniChem (51). The ChEMBL team supports the neglected-disease community, and now provides one-stop access to all data from the Medicines for Malaria Venture’s open-access MalariaBox (52) and other open-access malaria research efforts, including new high-value malaria and tuberculosis data sets. A version of ChEMBL has been built using only open-source software, and this has been made available as a virtual machine.

ChEBI, a resource for reference chemical structures, nomenclature and ontological classification, now offers a standalone tool for classifying compounds that resemble natural products. Several thousand natural products have been added to the database, and a new version of the SENECA tool, which helps to elucidate structures for natural products, has been implemented. ChEBI has also introduced new tools for searching (OntoQuery) and analysis (BiNChE) of information contained in the ChEBI Ontology.

## USER TRAINING

It is essential that our users can access EMBL-EBI’s data efficiently and get the most out of their own datasets when comparing them with the public record. To that end, EMBL-EBI provides an extensive user training programme (http://www.ebi.ac.uk/training), coordinated and funded centrally, but with input from all the resource teams. In turn, as training activities offer a unique interface between service developers and users, they are invaluable in the evolution of existing resources and the creation of new ones. EMBL-EBI’s diversifying community of users is reflected in its user training offering. The programme, courses and materials are created in response to user demand, and cover the full spectrum of EMBL-EBI’s activities.

Face-to-face training courses reach ∼4000 users a year—a small fraction of EMBL-EBI’s user community. Train online (http://www.ebi.ac.uk/training/online/), EMBL-EBI’s eLearning resource launched in 2011, supplies on-demand instruction to users the world over, in the form of free short courses designed for bench-based biologists. Quick tours provide an overview of each of the core data resources and show users where to go for more information. Introductory courses explain some of the important concepts behind the bioinformatics resources and introduce key subject areas, such as functional genomics. Walk-through courses provide a more in-depth exploration of a resource, structured as tutorials with use cases, guided examples and quizzes. Finally, video courses are based on some of our most popular face-to-face training courses. They provide video lectures and accompanying course materials.

Simply being able to discover relevant training courses is challenging for many life scientists, and EMBL-EBI’s training team has entered the realm of ‘training informatics’ through its involvement in the EMTRAIN project, which launched on-course® (http://www.on-course.eu)—a new resource for course seekers in the biomedical sciences—in June 2012 (53).

## CONCLUDING REMARKS

The foundations of EMBL-EBI’s data collection are comprehensive archival collections of biomolecular information, and our expanding and diversifying user base demands that we serve a growing number of specialized communities. To support research in the applied sciences, we must provide access to data from medical sequencing projects, with appropriately consented access to the data. EMBL-EBI is dedicated to remaining user-focused and developing interfaces and training opportunities that enable discovery in all areas of molecular biology. Our continuous interaction with our users is the driver that enables us to feed back to our developers, which, in turn, helps our services to remain relevant to our users’ needs.

## FUNDING

Funding for open access charge: Wellcome Trust.

*Conflict of interest statement*. None declared.

## APPENDIX 1

### EMBL-EBI’s mission

- To provide freely available data and bioinformatics services to all facets of the scientific community.
- To contribute to the advancement of biology through basic investigator-driven research.
- To provide advanced bioinformatics training to scientists at all levels.
- To help disseminate cutting-edge technologies to industry.
- To coordinate biological data provision throughout Europe.

### APPENDIX 2

#### EMBL-EBI’s principles of service provision

**Open**: Our data and tools are freely available, without restriction. The only exception is potentially identifiable human genetic information, for which access depends on research consent agreements.
**Compatible**: EMBL-EBI is a world leader in the development of global bioinformatics standards, which are key to data sharing.
**Comprehensive**: Thanks to our many data-sharing agreements, EMBL-EBI resources are comprehensive and up-to-date. We work with publishers to ensure that biological data must be placed in a public repository and cross-referenced in the relevant publication.
**Portable**: All of our data and many of our software systems can be downloaded and installed locally.
**High quality**: Our databases are enhanced through annotation: highly qualified biologists add value to databases by incorporating features of genes or proteins from other sources, and automated annotation is subjected to rigorous quality control.
